# Social participation of individuals with spinal injury using wheelchairs in rural Tanzania after peer training and entrepreneurial skills training

**DOI:** 10.4102/ajod.v12i0.975

**Published:** 2023-01-12

**Authors:** Annabelle de Serres-Lafontaine, Delphine Labbé, Charles S. Batcho, Lucy Norris, Krista L. Best

**Affiliations:** 1 Department of Rehabilitation, Faculty of Medicine, Université Laval, Quebec, Canada; 2 Center for Interdisciplinary Research in Rehabilitation and Social Integration (Cirris), Quebec, Canada; 3 Department of Disability and Human Development, University of Illinois at Chicago, Illinois, United States; 4 Motivation Charitable Trust, Bristol, United Kingdom

**Keywords:** social participation, manual wheelchair, spinal cord injury, peer training, accessibility, social support, advocacy

## Abstract

**Background:**

Individuals with spinal cord injury (SCI) in less-resourced settings reported barriers to community integration, including inaccessible rehabilitation services, restricted environments and limited social integration. Peer training and entrepreneurial skills training are provided by Motivation, a nonprofit organisation, and Moshi Cooperative University to enhance occupational engagement of individuals with SCI in less-resourced settings.

**Objective:**

This study aimed to explore the impact of peer training and entrepreneurial skills training on the social participation of individuals with SCI living in Tanzania.

**Method:**

Using a qualitative photovoice approach, 10 participants captured meaningful photos and provided captions according to five standardised questions (PHOTO technique) to convey their messages. Participants selected up to 34 photos that best illustrated their experiences in the community. A mixed inductive–deductive thematic analysis was guided by the International Classification of Functioning, Disability and Health.

**Results:**

Two interrelated themes emerged: (1) ‘influencing factors’, which revealed how participants’ inclusion in the community was influenced by their activities and personal and environmental factors and (2) ‘empowerment’, which highlighted participants’ desire to advocate and promote awareness of needs and hopes.

**Conclusion:**

Participants emphasised the importance of accessibility and equal opportunities. Whilst some were able to overcome obstacles, others experienced continued inaccessibility that inhibited meaningful occupations. Daily participation challenges of individuals with SCI in rural Tanzania were highlighted. Although the Motivation programmes were perceived to have powerful impacts on social participation, continued efforts and advocacy are needed to overcome accessibility issues and to meet the physical, psychological and social needs of Tanzanians living with SCI.

**Contribution:**

This article highlights the importance of accessibility and equal opportunities for individuals with disability living in rural Tanzania. Peer-training and entrepreneurial programs offer community-based rehabilitation services that were perceived by people with disabilities to have a powerful impact on social participation and vocation. However, continued efforts and advocacy are needed to meet the needs of Tanzanians living with spinal cord injury.

## Introduction

The World Health Organization (WHO) reported that 10% of people with disabilities (approximately 112 million people worldwide), need a wheelchair (WC) to enable mobility and social participation (Burrola-Mendez et al. 2018). Social participation is a multidimensional concept with various complementary definitions (Fudge Schormans 2014). The WHO describes social participation as a positive outcome and social determinant of health, considered both as a means for and as a goal of health equity (World Health Organization 2008). Others have added dimensions such as positive social interactions and receiving or contributing resources in society (Mars et al. 2008). Fundamentally, social participation has been defined as ‘a person’s involvement in activities that provide interaction with others in society or the community’ (Levasseur et al. 2010:2141–2149). Amongst people with spinal cord injuries (SCIs) who use WCs, social participation is often restricted because of environmental barriers (e.g. built environment), access to assistive technologies and services (e.g. WCs and WC skills training) and reduced opportunities (e.g. vocation) (Tsai et al. 2017). Social participation restrictions for people with disabilities are exacerbated in less-resourced setting (Fudge Schormans 2014).

Whilst it is recommended that WC service provision in less-resourced settings follows an eight-step process (i.e. referral, assessment, prescription, funding and ordering, product preparation, fitting, user training and a stage of maintenance, repairs, and follow-up), approximately 96 million people do not own a WC or their WC does not meet their needs (Smith, Sakakibara & Miller 2016; Toro, Eke & Pearlman 2016). This is especially true amongst those living in less-resourced settings, therefore undermining their right to personal mobility (Burrola-Mendez et al. 2018). Given the quality and fit of a mobility device plays an important role in successful social participation for people who have trouble walking, there is a critical need that people with SCIs have access to reliable and well-fit WCs and that they receive adequate training for safe and effective use (Magasi et al. 2018; Tsai et al. 2017).

Furthermore, despite governmental and global attempts to meet the assistive technology service provision needs of individuals with disabilities, these individuals face multiple barriers restricting or even preventing access to basic healthcare and rehabilitation services (World Health Organization 2015). In less-resourced settings such as in Tanzania, the common barriers restricting health service provision for individuals with physical disabilities (including people with SCIs who use WCs) are lack of education, discrimination, poverty, minimal access to resources and employment, maladapted or lack of assistive technology and architectural and social barriers (Tijm, Cornielje & Edusei Kwaku 2011). As a result, individuals in less-resourced settings commonly experience multiple unmet needs related to access to home and community-based services and restricted social participation (e.g. reduced mobility, access to health services, occupational engagement, education and other community activities). For example, in Ghana, the needs of people with physical disabilities were reported to be economic empowerment, counselling centres, affordable and quality rehabilitation, equal opportunities, education, accident prevention and shelters (Tijm et al. 2011).

According to the four domains of quality of life described by the WHO, individuals with SCIs face additional barriers to social participation, including physical health (e.g. pain, secondary conditions, dependance on medical aids), psychological (e.g. depressive symptomatology, negative feelings), social relationships (e.g. lack of social integration) and environment (e.g. physical safety and security, transport, home environment, health and social care and opportunities for leisure) (Barker et al. 2009; Kennedy et al. 2010; World Health Organization 2020). Whilst provision of WCs and other assistive technologies may help to overcome some of the barriers, WC provision alone does not address all critical aspects of WC service provision, nor will it ensure meaningful engagement in occupational and social activities or quality of life, especially in less-resourced settings (Norris 2017).

To help bridge this gap, a nonprofit organisation, Motivation (https://www.motivation.org.uk), has developed peer-support programmes and targeted skills training programmes to address the needs of individuals with SCIs and other disabilities. The main goal of peer training is for WC users (i.e. peer trainers) to provide other WC users with relevant knowledge and skills (Norris 2017) in an effort to improve independence, quality of life, access to services and occupational engagement in less-resourced settings (Norris 2017). The Motivation Peer Training (MPT) programme included three core elements including improving WC mobility skills (e.g. pushing over rough ground), increasing knowledge on health issues and well-being (e.g. understanding the main causes of disability) and understanding and advocating for their rights. Previous research also suggests that MPT can reduce the feeling of social isolation and improve perspective on ability to access recreational activities (Rushton et al. 2011). Therefore, in a search for equal opportunities, inclusion, respect of human rights, vital information on disability rights and advocacy, support mechanisms may be provided by Motivation for WC users in less-resourced settings (Norris 2017). To enhance reach to more people in need of quality training across less-resourced settings, the organisation has implemented a standardised approach to training, which has reached more than 11 500 WC users in 22 countries to date (Norris 2017).

Peer-led approaches have been reported as a useful method for providing WC training in less-resourced settings (Best et al. 2018; Norris 2017) and appear to be quite influential for people with SCIs (Kirby 2017). In fact, local researchers in Nairobi, Kenya and Manila, Philippines interviewed 48 WC users, with even distribution amongst those who had and had not received peer-led training when they received their WC (Williams et al. 2017). Qualitative findings revealed four fundamental themes regarding the implementation of WC services, including physical environmental barriers, the need for multiple WCs to improve access, perceived social stigma and the importance of peer support (Williams et al. 2017). It was shown that peer support networks provided shared life experiences, reduced feelings of social isolation and were perceived as an important source of knowledge, skill acquisition (e.g. related to WC use and maintenance, employment and income generation), developing a sense of belonging, emotional support and well-being (Norris 2017; Standal & Jespersen 2008; Williams et al. 2017). Other studies added that a peer-to-peer approach induced positive behaviour changes, as peers are thought to be more credible than able-bodied people when it comes to demonstrating manual WC (MWC) techniques (Rushton et al. 2011). In fact, self-efficacy and self-esteem related to MWC use, community participation and mobility within living environments increased after peer support (Best et al. 2016; Sakakibara et al. 2013, 2014).

In high-resourced settings, peer-led MWC training has been shown to be feasible and effective for improving MWC skills capacity, performance and satisfaction amongst adults and older adults (Best et al. 2018). Without follow-up services or proper formal WC training in less-resourced settings, MPT may be a key strategy to increase WC service efficiencies (Williams et al. 2017). However, descriptive data regarding the implementation of WC services in less-resourced settings and the perceptions of WC users about whether those services are meeting their needs remains largely absent (Smith et al. 2016).

The purpose of this study was to explore social participation (including barriers and facilitators) of individuals with SCIs using WCs in rural Tanzania after completion of the MPT and entrepreneurial skills training (EST) programmes. Understanding perspectives of individuals with SCIs who use WCs regarding accessibility and community inclusion may inform refinements to the MPT and EST programmes, including approaches to peer mentorship in a less-resourced settings. More importantly, it will show the current needs of people with SCIs who use WCs, which could inform policy changes needed to enhance social participation.

## Method

### Motivation, peer training and entrepreneurial skills training

Before this study started, Motivation and Moshi Cooperative University provided MPT (delivered by peer trainers who use MWC) and EST (delivered by trainers from the Moshi Cooperative University, Tanzania), followed by provision of assets to start a small business (e.g. chicken coop, small shop). All Motivation staff were previously trained to deliver the MPT programme. Firstly, the MPT home visits consisted of 16 one-hour sessions of peer-led WC training in the participants’ homes. Peers covered various topics from the MPT training package, including disability awareness (e.g. knowledge on SCIs and other disabilities, rights and advocacy), self-care and health (e.g. skin, bladder and bowel care and sexuality) and mobility (e.g. WC skills, transfers and sports). The package was created to supplement the support and medical services that individuals with disabilities were already receiving. It was revised in 2016, based on feedback from trainers and trainees. Since then, it has expanded to include additional WC skills and sessions on HIV, AIDS and appropriate WCs (Norris 2017:350). Secondly, EST consisted of a three day course, which included sessions on disability and economic development, introduction to entrepreneurship and setting up savings and support groups.

### Design

A phenomenological qualitative design was used to explore all dimensions of social participation as perceived by the participants. The photovoice method was used to explore social participation through lived experiences, current social inclusion, occupational engagement and access to home and community-based services. Photovoice, originally developed to empower marginalised groups (Wang & Burris 1997), is a participatory community-based approach allowing marginalised groups to take and present photos of issues and concerns in their daily lives and discuss them collectively with a group of individuals with shared life experiences in focus groups and during an exhibition to promote awareness and change. Photographs allow people to present their personal perspectives in their own voices, in a position of advocacy that could influence policy, decision makers and the way social concerns are understood and addressed (Liebenberg 2018; Tijm et al. 2011). In fact, it was recommended that photovoice be considered more often to capture perspectives about living with physical disabilities in rural communities in less-resourced settings (Dassah, Aldersey & Norman 2017). Conducting photovoice in a rural, less-resourced setting may increase our knowledge and understanding of the challenges individuals with physical disabilities may be facing (Dassah et al. 2017). Photovoice has also been described as particularly efficient when sharing findings about person–environment interaction in a more compelling manner than narrative data from interviews and focus groups (Catalani & Minkler 2010).

### Participants and recruitment

All WC users with SCIs who previously completed 1 – 3 MPT home visits and the EST in Tanzania were invited to participate in this study. A convenience sample was recruited by the research coordinator in Tanzania, including those who met the inclusion criteria: 18 years of age or older, diagnosed with a SCI and using a MWC.

### Data collection

Within 6 – 12 months after the completion of the MPT and EST programmes, participants completed a sociodemographic questionnaire and a two hour workshop in Swahili (the local language) to prepare them for the study (education about photovoice technique, participation and empowerment). More specifically, they received a lesson in basic photography and were introduced to photovoice (i.e. the process of taking pictures and writing small captions to help capture the meaning of the image) (Palibroda et al. 2009). Cameras were provided to participants by Motivation for the purposes of the research study. Participants were also trained in ethics, privacy, safety and photo selection (Evans-Agnew & Rosemberg 2016). For example, they discussed ethical issues and the use of digital cameras (e.g. respect and privacy, safety of participants and responsibility) (Wang & Redwood-Jones 2001). The training focused on individual empowerment by including an ‘I can do it’ attitude affirmation, with the aim of highlighting the capabilities of the participants.

To explore their social participation in their community, participants were instructed to take meaningful photographs of their everyday activities (e.g. people, places or things) over a period of four weeks (photos were taken between June 2019 and September 2019). Afterwards, participants selected 34 pictures that best illustrated their experiences in the community and portrayed how they felt facilitated or inhibited in their chosen social participations and occupation engagements. To reach a deeper understanding of the selected pictures, the PHOTO technique was used to consider participants’ realities through their own eyes and personal perspectives. This technique was designed to facilitate discussion of selected photographs through five standardised key questions:

- Describe your photo.
- What is happening in your picture?
- Why did you take a picture of this?
- What does this picture tell us about your life?
- How can this picture provide opportunities for us to improve life? (Wang et al. 2004)

Participants then contextualised their photographs by telling stories about what the picture means to them and how it reflects their personal or community strengths and issues. Indeed, the VOICE acronym (i.e. voicing our individual and collective experience) was used to explore important issues and to remind participants to think not only about their own life conditions but also about shared life events and conditions that could result in social change (Liebenberg 2018). All staff were trained to facilitate the photovoice workshops.

### Data analysis

Sociodemographic information and qualitative data (i.e. PHOTO technique answers for each selected photograph) were collected and translated from Swahili into English. Thematic analyses of the photos and captions using a mixed deductive–inductive approach were conducted by Annabelle de Serres-Lafontaine. Validation of codes by the research team was conducted midway and at the end of the analyses process. Firstly, data were coded deductively according to the International Classification of Functioning, Disability and Health (ICF) (World Health Organization 2021). Organisation and conceptualisation within the ICF comprise two parts, such that the first part contains components relating to functioning and disability (i.e. ‘Body Functions and Structures’ and ‘Activities and Participation’) and the second part contains components related to contextual factors (Environmental and Personal Factors). These components are denoted by prefixes in each code: ‘b’ for body functions; ‘s’ for body structures; ‘d’ for activities and participation; and ‘e’ for environmental factors (World Health Organization 2007). After initial themes were explored, an inductive approach was used to explore emergent themes.

### Ethical considerations

Ethical approval for this study was obtained from the Comité d’éthique de la recherche sectoriel en réadaptation et intégration sociale, CIUSSS de la Capitale-Nationale (#2020-1810, RIS_ jusqu’au). Informed consent forms were translated from English to Swahili by a research coordination in Tanzania and signed. Written consent was also obtained from any people who were included in the photos (with faces blurred to ensure privacy).

## Results

Ten participants ranged in age from 30 to 45 years; five were female and six were married. All had received MPT and EST from Motivation and all spoke Swahili as their first language.

Two interrelated themes emerged from the data analyses, including: (1) ‘influencing factors’, which revealed participants’ community integration was influenced by their activities and participation, as well as personal and environmental factors; and (2) ‘empowerment’, which explained participants’ needs of advocacy and awareness and their hopes for future changes and a meaningful life.

The themes are presented with an inductive term, followed by the associated ICF labels and corresponding codes in parenthesis. In addition, quotes from the PHOTO technique and some of the participants’ shared photographs identified with the participants’ number (in brackets) were used to illustrate the themes.

### Theme 1: ‘Influencing factors’

#### Activities and participation (d: activities and participation).

**FIGURE 1:**
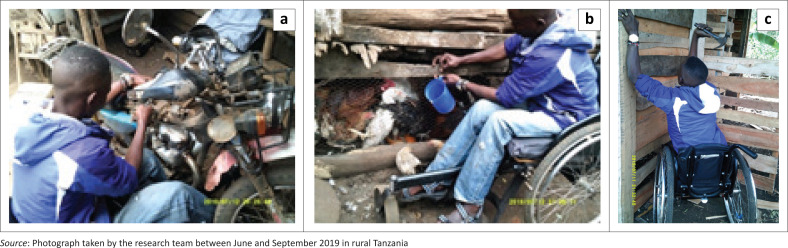
Engaging in accessible livelihood: (a) Repairing a motorcycle; (b) Having a chicken coop; (c) Building a chicken coop.

Livelihoods and activities of daily living were discussed in this subtheme. Some participants mentioned different livelihoods (d840–d859: work and employment) that they were able to do, such as having an accessible chicken coop or repairing motorcycles (Figure 1). Those seemed to be ‘one of the many ways for generating income for the wheelchair users’ (Participant 10, male, SCI). However, other participants mentioned that the lack of accessibility of some places limits their livelihoods opportunities. For example, one said: ‘if the (marketplace’s accessibility) was improved, I could do my own business as well, in addition to buying things there’ (Participant 4, female, SCI). As for one of their main activities of daily living (d5300: regulating urination), in terms of their self-care related to SCI, participants mentioned they have access to a doctor who prescribes them a catheter, but they face multiple challenges in accessing urinary continence management products (UCMPs) and information on urinary contingency management. Also, considering that ‘the hygiene and cleanliness are not appropriate in the health facilities’ (Participant 1, female, SCI), the sterility of catheters and UCMPs was questionable.

#### Personal factors (body functions: b)

**FIGURE 2:**
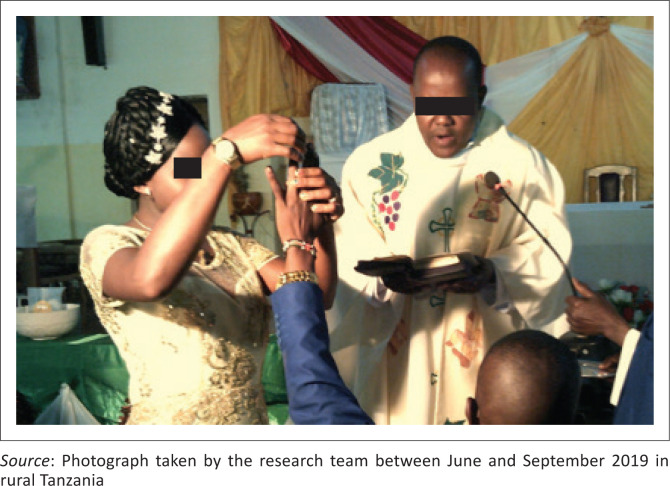
Getting married and feeling confident.

Participants’ self-efficacy and self-esteem were invoked in this subtheme. Indeed, they expressed the importance of a good sense of self-efficacy, that individuals with disabilities should be active and believe in their own capacities. For instance, one participant showed a picture of himself building a chicken coop and mentioned that ‘every wheelchair user should be able to see their own abilities’ (Participant 10, male, SCI). As for the sense of self-esteem, one participant expressed that it is ‘one of the many challenges people with disabilities face’ (Participant 10, male, SCI). He showed himself at his wedding ceremony and expressed that people with incapacities can also get married and feel confident in their own skin (Figure 2). He expressed, ‘trust your ability in building your own self-esteem’ (Participant 10, male, SCI).

#### Environmental factors (environmental factors: e).

Participants described aspects of their physical environment (i.e. assistive technologies, transportation and access to places and facilities), as well as their social support.

**FIGURE 3:**
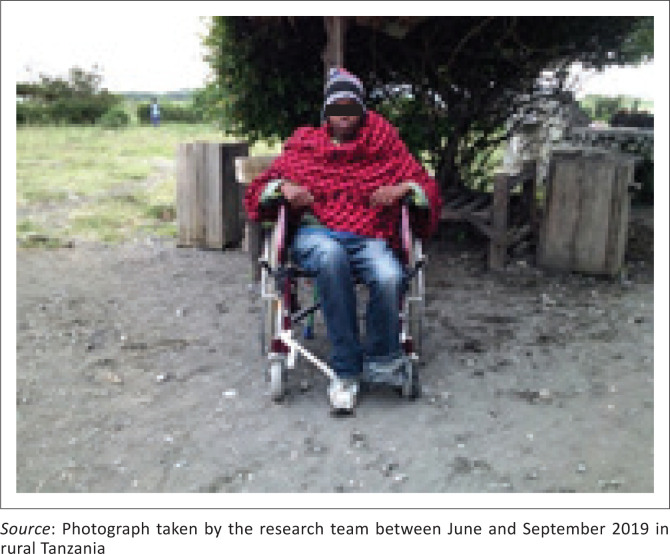
Receiving maladapted assistive technologies.

**FIGURE 4:**
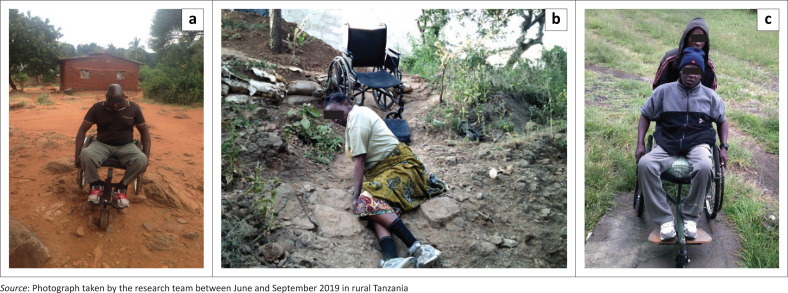
Moving on rocky roads and steep inclines: (a) moving on rocky and uneven roads; (b) moving on steep inclines to access the community; (c) needing human help on steep inclines.

Some participants mentioned having assistive technologies (e1201: assistive products and technology for personal indoor and outdoor mobility and transportation) that were not adapted and missing certain parts (Figure 3). One said: ‘the wheelchair is not appropriate. There is no footrest, it is bigger than needed and the tire went out’ (Participant 8, male, SCI). Moreover, a lot of participants reported obstacles in the urban and rural environment (e160: products and technology of land development), which limited their ability to get around. In fact, they put emphasis on how bad the roads are in their village: they described them as rough, uneven and rocky, with steep slopes and no sidewalks (Figure 4). This interfered with or even prevented independent transportation with their WC and thus hindered their access to basic needs (e.g. water) and infrastructures. In addition, it made it difficult for them to propel their WC on the rough roads and move safely and easily. One participant expressed, ‘there is no pedestrian pathway in our community. The [*road*] is not friendly to the people with disabilities in our community’ (Participant 5, female, SCI). Another showed himself on a steep hill and explained, ‘the picture is showing how steep the incline is – a wheelchair user cannot move independently. I need to be assisted’ (Participant 8, male, SCI).

**FIGURE 5:**
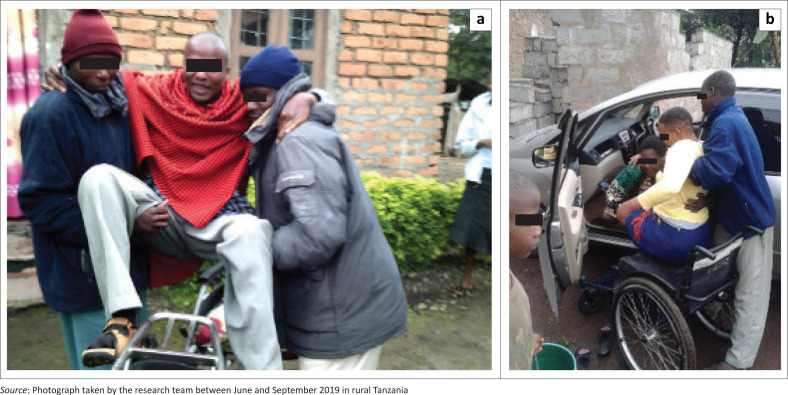
Transferring from the wheelchair to transportation inclines: (a) needing human help to get onto a motorcycle; (b) needing human help getting in a car.

Furthermore, participants identified barriers accessing transportation services (d470: using transportation), whether a bus, a motorcycle or a car (Figure 5). As one participant boarding a bus mentioned, ‘the doors of public transport vehicles are not easy to go through if you have a disability’ (Participant 5, female, SCI). Not only it is difficult to use public transportation, but it also takes more space and generates more expenses, as another participant explained: ‘When boarding the bigger bus, I am using two seats to transfer my wheelchair and paying for both’ (Participant 6, male, SCI). Given that ‘the availability of public transport is very rare’ (Participant 3, male, SCI), some participants highlighted that they sometimes needed to use a motorcycle, which they found dangerous, difficult to access and to stay in balance, and not very useful because they could not carry their WC.

**FIGURE 6:**
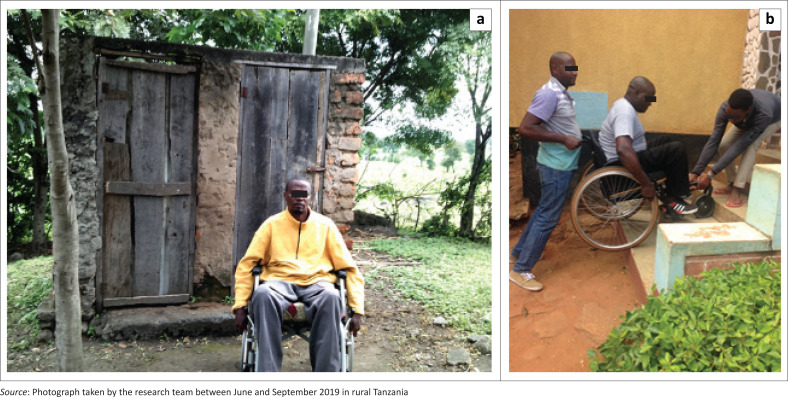
Facing accessibility issues and depending on others’ help to overcome the: (a) uneven entrance hindering access to a public bathroom; (b) needing human help accessing church because of stairs at the entrance.

Participants highlighted that most places and facilities (e150: design, construction and building products and technology of buildings for public use) are inaccessible to individuals with disabilities for various reasons such as the presence of stairs, steps, narrow doors and uneven entrances and the absence of ramps (Figure 6). Those barriers limited or even prevented them from moving around independently and accessing the entrance and upper floors of various buildings. As a result, these obstacles impeded participants’ access to services and social participation in different daily activities, such as grocery shopping and selling in markets, receiving basic pharmaceutical needs and having confidential appointments in medical clinics, going to governmental offices and school as well as going to church. For example, participants mentioned, ‘markets are not accessible. The seller is asking me what I want and I’m staying far from her’ (Participant 4, female, SCI). A participant also presented a picture of the school and expressed:

‘[*T*]he picture is showing an inaccessible classroom with steps and a narrow door. The classroom does not have ramps so a student with a disability cannot have access to the school.’ (Participant 8, male, SCI)

Participants also mentioned that even home environments (e155: design, construction and building products and technology of buildings for private use) were ‘[…] not friendly for wheelchair users’ (Participant 3, male, SCI). In fact, similar barriers as those previously mentioned were described when accessing private houses or even their own residences, such as stairs or the absence of ramps. One participant talked about the challenges he was facing whilst visiting his relatives and how it decreased his independence: ‘my relatives are carrying me out after the visit’ (Participant 3, male, SCI). Another mentioned the presence of stairs when he explained ‘I’m waiting for someone to take me downstairs. My house is not accessible’ (Participant 2, male, SCI). In addition, a participant mentioned that the bathrooms are inappropriate: ‘not only are they inaccessible but the toilet is also narrow and without grab bars’; he added that it ‘(…) does not have a cover and the edges are sharp’ (Participant 8, male, SCI). which can be dangerous.

Regarding participants’ social support (e3: support and relationships), they all expressed that they needed human assistance to facilitate their social participation, given the multiple barriers in their physical environment (Figure 6). For instance, WC users needed help from one or more individuals to carry heavy objects (e.g. big bottles of water) for their basic needs or move about on the rocky and uneven roads. One person explained how ‘the church premises are not accessible. People are carrying me to the church, [*and later I am*] getting down the stairs from the church alone’ (Participant 6, male, SCI). However, participants were not always accompanied by relatives or citizens, so they often had to transfer out of their WC to climb up and down the stairs on their own or wait for someone to come by: ‘I am waiting for assistance to take me to the pharmacy’ (Participant 6, male, SCI). Also, someone added that she felt very vulnerable and dependent on others because of her disability:

‘The environment is inaccessible in inhabited areas: I am getting out of my wheelchair to crawl to the other side of the hill to seek help and access the community.’ (Participant 5, female, SCI)

Finally, some participants expressed communication difficulties during transfers affecting themselves and helpers: ‘the issue of communication when you are carrying the person with a SCI is important, you have to communicate properly’ (Participant 2 male, SCI).

### Theme 2: ‘Empowerment’

#### Advocacy and awareness

Participants were unanimous in voicing their needs and advocating for equal rights for all (d940: human rights; d950: political life and citizenship; e5 services, systems and policies). They emphasised that the pictures they took were for an educational purpose and might help the entire community understand disabilities and consider individuals with incapacities as whole and complete individuals, as well as raise their awareness about the issues they are facing daily. When asked about the reason they took their pictures, some participants responded, ‘to educate the community about inclusiveness. The community is not conscious about disability issues’ (Participant 5, female, SCI and Participant 8, female, SCI). Another person said they took their pictures ‘to express the challenges people with disabilities are facing’ (Participant 7, female, SCI). Others focused on specific actors in the community (i.e. public transport providers, religious authorities, landlords, education departments, local governments and officials) for which they advocated for the accessibility of the services they provide to the community.

Indeed, participants brought awareness on the importance of having accessible infrastructures and vehicles as well as renovating nonaccessible ones to minimise social participation barriers and optimise accessibility to services. As one person emphasised:

‘[*A*]ll human beings have equal rights. If we advocate for changes regarding accessibility issues, the community will be changed. We have to increase the level of independence of people with disabilities […].’ (Participant 9, female, SCI)

For instance, they mentioned that building accessible bridges, sidewalks, pathways, roads, ramps and wider doors would be beneficial. Some participants added that:

‘[*T*]he public transport provider should consider (people with disabilities) and adapt cars to be accessible. The community should have cars instead of motorcycles as public transportation options so there would be less accidents.’ (Participant 1, female, SCI and Participant 5, female, SCI)

However, another participant explained that when building something new, the government does not prioritise accessibility for all even though they should consider and provide for the needs of the entire population, including individuals with disabilities, when he stated:

‘[*I*]n my community, they are not giving priority to people with disabilities’ choices. When we are constructing a building in our community, we have to make sure it is accessible. We are supposed to build friendly and accessible environments.’ (Participant 9, female, SCI)

Others who did not have accessible residences added that their landlords were not conscious about accessibility issues and landlords were not willing to pay for modifications (e.g. installation of ramps). Therefore, to make their community evolve in a positive way, participants advocated for being included in decision-making when building new infrastructure, ‘I am asking the government to build the infrastructure and work with the community. Once we are constructing anything, we have to take ramps into consideration and accessibility issues’ (Participant 5, female, SCI and Participant 9, female, SCI).

Furthermore, with a more accessible environment comes better access to services and meaningful occupations. For instance, one participant expressed:

‘[*W*]e need to express to the officials how important it is for the markets to be accessible. I want to go to the market and choose for myself what to buy, and I want to tell the community I have the right to do so.’ (Participant 4, female, SCI)

Participants raised awareness about ‘the challenges with livelihoods that people with disabilities are facing in securing basic needs’ (Participant 3, male, SCI) and ‘how important it is to help people with any special needs in accessing livelihoods’ (Participant 3, male, SCI). They wanted the community to be aware of the unavailability of UCMPs, the barriers to getting water, their desire to have more confidentiality when they speak to the medical staff, the services that are only available on upper floors, the inaccessible toilets either in public places or private houses and their obligation to pay for two seats on the bus, amongst other things that were all influencing factors for their social participation in the community.

#### Hope for the future

**FIGURE 7:**
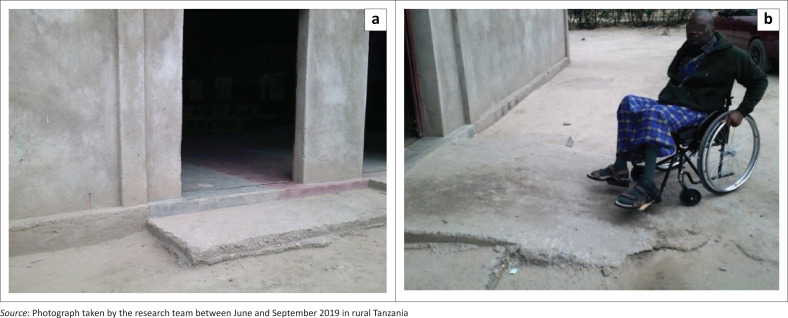
Building a ramp at the church’s entrance: (a) before and (b) after.

The WC users who experienced help from others felt that they were able to engage in more activities in their daily lives. Moreover, participants also perceived that meeting people who were curious about their disability or their WC provided them with an opportunity to educate others about their reality. For instance, one participant talked about SCI as part of the community awareness outreach programme at the radio station. Moreover, a participant emphasised that ‘[…] people with disabilities […] should not be losing hope of having an enjoyable life’ (Participant 10, male, SCI), as he was still able to have a fulfilling and happy life with his spouse, who shared his hope for future changes in the community. Indeed, after the participant sought and requested independent access to the church, a pastor proceeded with the construction of a ramp at the entrance (Figure 7). This participant added that this change should motivate similar initiatives from church administration or the community at large.

## Discussion

A variety of factors such as economic, social and personal circumstances (e.g. having a physical disability, living in a remote area) influenced perceived equality and fair access to resources. Therefore, the experiences of social participation for individuals with SCIs who used WCs in rural Tanzania varied according to personal circumstances. Overall, accessibility challenges and advocacy for needs and rights were the predominant issues raised by participants. Participants described limited opportunities to contribute meaningfully to the community and for social interaction and perceived restricted access to home-based and community-based services (i.e. important factors of social participation) (Best et al. 2022; Mars et al. 2008:1298–1308).

### Accessibility issues

Participants in this study revealed multiple environmental barriers to mobility, community participation and securing basic needs, including inaccessible infrastructures (e.g. school, pharmacy, house), rough roads and limited transportation services (Barclay et al. 2016). In studies involving individuals with SCIs in less-resourced settings, similar barriers to social participation and independence were highlighted and shown to increase risks of poverty, social isolation, mental health issues and unemployment (Löfvenmark et al. 2016; Maclachlan 2012). The MPT programme facilitated some participants to better contribute to or receive resources from their community, which enhanced their social participation (Mars et al. 2008:1298–1308). For example, topics during MPT (i.e. self-care and health, mobility) enhanced participants’ knowledge about their condition and WC skills, specifically helping them to better target their health service needs. Furthermore, EST gave some of the participants the skills and motivation to start a livelihood (e.g. chicken coop). However, whilst a few participants were able to overcome the obstacles (e.g. attending church, seeing a doctor, building a chicken coop), most of them experienced continued inaccessibility that inhibited meaningful occupations (e.g. buying and selling at the local market, functioning independently in their own home, entering a car), confirming the need to move towards an accessible community to enhance well-being and inclusion (Löfvenmark et al. 2016).

When discussing social participation in their community, participants described frequent feelings of vulnerability because they depended on others to access infrastructures and to move around. In fact, previous research supports that many individuals with disabilities depend on others to help with mobility and transfers, due to physical dependence and poor social integration, which can decrease overall quality of life (van der Woude et al. 2013). Moreover, restricted mobility and inadequate WC fitting and seating are associated with various health issues (e.g. pressure injuries, falls, overuse or repetitive strain injuries, postural abnormalities, restricted breathing and a limited range of motion) (Sprigle 2014).

These environmental barriers constitute an important matter considering that in Tanzania, amongst other issues raised in less-resourced environments, there are already unequal education opportunities for children with special needs (UNICEF), lack of accessible, hygienic and adequate health services (Swere 2016) and lack of access to clean drinking water, compelling most residents to rely on different sources of water at a distance from home (Smiley 2013). However, as a result of the various environmental barriers being presented to government, policies now state that all new buildings should be made accessible. Nevertheless, there are currently no laws or government funds in place to reinforce the policies to ensure buildings are accessible (Norris 2017).

### Advocating for needs and equal rights

Through their photographs, participants advocated for their needs and raised awareness about SCI and disability issues to minimise the judgements and social stigma associated with disability, which is a worldwide issue (Barclay et al. 2016). Indeed, although their rights are protected in national law and legislation, people with disabilities are less likely to be included in political activities, leadership roles, education, gainful employment or access to financial resources and support networks (e.g. saving cooperatives) (Tijm et al. 2011; Virendrakumar et al. 2018). These unequal opportunities can further exacerbate negative psychological impact, given that 20% – 30% of people with SCI show clinically significant symptoms of depression that are substantially higher than the general population (Norris 2017). To address this inequity, participants expressed the need to be included in governmental decision-making when building new infrastructures or establishing new policies, to ensure inclusion and to educate the community. Although the session on disability awareness during MPT allowed some participants to educate others through meaningful and positive interactions, which may have enhanced their social participation (Mars et al. 2008), continued efforts to raise awareness on disabilities and to overcome accessibility issues are needed to further enhance attainment of community integration and to respect their rights (Löfvenmark et al. 2016).

Helping the entire community understand the reality of living with a disability may be the genesis of promising changes, enabling equal opportunities, positive social attitudes, greater independence and social participation amongst participants (Löfvenmark et al. 2016), thus a better quality of life (Barker et al. 2009). Indeed, studies show that empowering individuals with disabilities to better advocate for their rights (e.g. to employment and education) and to be included in their communities could improve confidence in their abilities to access various occupations, higher education and employment when given the right knowledge and skills for independent living (Norris 2017). As some participants said following the peer training, it is possible for people with disabilities such as themselves to stay active, have good self-esteem, generate income and most of all, live a fulfilling life. Motivation is even working on integrating government advocacy (e.g. lobbying the Ministry of Health for the course to be included in health institutions and including it in the School of Nursing syllabus) with programme-planning of peer-training in Tanzania. This could promote institutional change and improve government practices, raise awareness on disabilities and reduce costs (Norris 2017). After all, to ensure the application of human rights for all, considering that disability is partly caused by social conditions, the community has a collective responsibility to compensate for environmental barriers previously stated and provide for full participation of individuals with disabilities in all areas of social life (Solli & Barbosa Da Silva 2012).

### The international classification of functioning, disability and health as an organising framework

This article provides some insight into the application of the ICF to explore social participation and inclusion of individuals with SCIs in rural Tanzania. In fact, some subthemes originating from the thematic analysis (i.e. activities and participation, environmental factors and advocacy and awareness) were explicitly related to ICF domains and identified by participants as elements that daily influence their community engagement (World Health Organization 2021). However, although personal factors in the ICF did include psychological components such as self-esteem and self-efficacy (Geyh et al. 2012), the conceptual framework did not fully capture the depth of information required to understand the affective components of the person. Indeed, some subthemes (i.e. personal factors, including participants’ self-esteem and self-efficacy, as well as hope for the future) were not explicitly portrayed and described in the ICF, yet these concepts influence individual daily occupational engagement (e.g. leisure, livelihoods), relationships, choices and desires (e.g. pursuing higher education) (Ravenek et al. 2013).

### Reflections on the motivation programme: Recommendations for the future

Based on participants’ experiences and participation after MPT, recommendations may be made for future practice. The MPT peer-training courses focused largely on WC skills and using the device (e.g. WC transfers, basic WC mobility, WC maintenance, assistance and assistive devices). The MPT programme may be enhanced by following the WHO 8-step service provision guidelines (Norris 2017). Moreover, MPT could expand their focus to target the development of knowledge and skills to better communicate and advocate for their personal needs when receiving mobility assistance. In this way, the self-management of health needs may be promoted (e.g. postural needs, facilitate safer mobility and transfers), which would ultimately lead to greater independence, social participation and livelihoods (Norris 2017). Furthermore, to better empower individuals with SCI, MPT sessions could facilitate valuable role-modelling, provide necessary tools (e.g. coping and action planning tools) and resources for social support and modify practices according to disability rights and advocacy to facilitate personal change and educate others (Löfvenmark et al. 2016). At an institutional level, peer trainers could even run lobbying activities (e.g. include people with disabilities in sport events, raise awareness on the International Day of Disability).

In line with the future, participants highlighted the importance of building their own self-esteem and self-efficacy regardless of others’ judgements and remaining optimistic about living a happy life in an inclusive community, which can all be strengthened through peer training. In fact, some studies highlight that personal factors and goals should be seen as central in the ICF (Solli & Barbosa Da Silva 2012). Similarly, other studies explored the need to encompass a biopsychosocial perspective of people’s activities and participation within their social systems (e.g. individual, family, community). Such a holistic approach could facilitate an understanding of how biological, physical, psychological and social factors can influence one another, contribute to functioning and well-being and be modulated by the environment (e.g. others’ support and attitudes, services and policies) (Ravenek et al. 2013; Solli & Barbosa Da Silva 2012:277–294). Given that peer-training can improve WC skills, confidence, self-esteem and self-efficacy and facilitate achievement of individually set goals and aspirations (Best et al. 2016; Worobey et al. 2016), this supports the need to integrate affective components of the person into assistive technology interventions and peer training programmes such as MPT.

## Limitations

As a result of the small sample size and the exploratory nature of photovoice methodology in a less-resourced setting, the findings of this study may not be generalisable to all people with SCIs who use WCs. In addition, details about the level of SCI were not obtained. Therefore, it was not possible to comment about level of functioning or mobility limitations on social participation. The completion of the Motivation programme and the involvement of the organisation in delivering the photovoice preparatory workshop may also have imposed some bias on the photos the participants took and the photos they chose to discuss, which remains subjective and therefore may lack replicability. However, although these results are specific to participants in rural Tanzania, similar challenges that people with disabilities face daily have been identified in other less-resourced settings, suggesting some transferability of the results in similar contexts. The study materials were prepared in English and informally translated to Swahili. Given there was no standardised translation method followed (e.g. forward-backward translations), it is possible that cultural nuances were not adequately considered. However, the questions in the PHOTO methods are basic and not related to any geographical or cultural nuances. Finally, the ability of all participants to use a camera may differ, even though they received training beforehand. This may have affected the quality of the photographs that participants were able to take and the consequent data collected.

## Conclusion

This study provided evidence about the social participation and occupational engagement of people with SCIs using MWCs in rural Tanzania, underlining the importance of accessible environments, access to services, social support, psychological well-being and inclusion of individuals with disabilities in the community as equals. Motivation Peer Training and EST had meaningful impacts on the lives of the participants, helping them improve their MWC skills, engage in meaningful livelihoods contributing to the community and to educate others through positive social interactions. However, remaining barriers emphasised the need for continued efforts to improve accessibility of infrastructure and community-based services, to empower people with disabilities, to enhance advocacy and to create awareness about daily challenges, which may be targeted through peer-training programmes. Findings from this study suggest target areas to enhance social participation and promote community integration of individuals with disabilities in less-resourced settings. Future research may consider refining peer-training approaches and targeting policymakers.
